# Immunoresolvents signaling molecules at intersection between the brain and immune system

**DOI:** 10.1016/j.coi.2017.10.007

**Published:** 2018-02

**Authors:** Jesmond Dalli, Charles N Serhan

**Affiliations:** 1 Lipid Mediator Unit, William Harvey Research Institute, Bart's and the London School of Medicine, Queen Mary University of London, London EC1M 6BQ, United Kingdom; 2 Center for Experimental Therapeutics and Reperfusion Injury, Department of Anesthesia, Perioperative and Pain Medicine, Building for Transformative Medicine, Brigham and Women's Hospital and Harvard Medical School, Boston, MA 02115, United States

## Abstract

Understanding mechanisms that control immunity is central in the quest to gain insights into the etiopathology of many of the diseases that afflict modern societies. New results implicate the nervous system as a central player in controlling many aspects of both the innate and adaptive arms of the immune response. Furthermore it is now well appreciated that a novel group of autacoids termed as specialized proresolving mediators, which are enzymatically produced from essential fatty acids, orchestrate the immune response promoting the termination of inflammation as well as tissue repair and regeneration. The present brief review discusses evidence for the crosstalk between the nervous system and leukocytes in regulating SPM production. We will also discuss the impact that this has on controlling tissue resolution tone and the resolution of both infectious and sterile inflammation.

## Highlights

• The vagus nerve regulates ILC-3 and macrophage trafficking to the peritoneum.
• Vagotomy reduces peritoneal PCTR concentrations and alters tissue resolution tone.
• Resection of the vagus nerve impairs resolution responses to sterile and infectious insults.

**Current Opinion in Immunology** 2018, **50**:48–54

This review comes from a themed issue on **Innate immunity**

Edited by **Gwendalyn J Randolph**

For a complete overview see the Issue and the Editorial

Available online 16th November 2017

**https://doi.org/10.1016/j.coi.2017.10.007**

0952-7915/© 2017 The Authors. Published by Elsevier Ltd. This is an open access article under the CC BY license (http://creativecommons.org/licenses/by/4.0/).

## Introduction

The nervous system integrates signals from the periphery to ensure homeostatic control and host survival. Increasing evidence demonstrates that in addition to regulating sensory and motor functions the nervous system is also essential in regulating the host immune response [1]. This crosstalk between the immune and nervous systems is a primordial defense mechanism where, for example, in the nematode *Caenobacter elegans* the nervous system is central in controlling innate immunity via the modulation of the non-canonical unfolded protein response [2]. In mammals the nervous system is also important in controlling both the innate and adaptive arms of the immune responses [1, 3, 4]. Recent studies demonstrate that the vagus nerve orchestrates host responses to both sterile, including sterile peritonitis [5], arthritis [6], colitis [7] and cancer [4] as well as infectious insults, for example, polymicrobial sepsis [8] and *Escherichia coli* infections [3]. These observations have been extended to humans where in patients with arthritis stimulation of the vagus system leads to a significant reduction in disease activity and in circulating markers of inflammation [9].

Efforts to identify signals that control the termination of inflammation recently uncovered a genus of mediators that are produced via the stereoselective conversion of essential fatty acids. These autacoids activate cognate receptors and regulate the biological actions of both innate and adaptive immune cells [10]. Given their potent actions and unique structures these mediators are coined as specialized pro-resolving mediators (SPM). This super-family of mediators is composed of four main families: the arachidonic acid derived lipoxins, the eicosapentaenoic acid (EPA), *n*−3 docosapentaenoic acid (DPA) and docosahexaenoic acid (DHA)-derived resolvins (Rv) and the *n*−3 DPA and DHA-derived protectins (PD) and maresins (MaR) (see [11, 12] for a detailed review of their biosynthetic pathways). The production of these mediators is regulated in both a temporal and tissue dependent manner. Recent studies demonstrate that the biological actions of RvD1 (see Table 1 for complete stereochemistry), a DHA-derived SPM, are additive to those of antibiotics reducing the required doses to clear both gram positive and negative infections [13]. These mediators also regulate tissue repair and regeneration by controlling leukocyte trafficking, phenotype, the expression of genes that are involved in the regeneration of damaged tissues and the re-establishment of barrier function [14, 15, 16]. Impaired LXA_4_ production is associated with dysregulated T-cell response in dry eye disease [17]. RvD1, RvD2 and MaR1 also regulate T-cell phenotype, down-regulating the production of effector cytokines, including IFNγ, in both CD4 and CD8 positive T-cells. They also promote the expression of FoxP3 in CD4 cells [18]. The RvD series precursor 17-HDHA regulates B-cell responses to viral infections, up-regulating antibody production and protecting against influenza infections [19] and pain [20, 21]. The EPA derived RvE1 regulates the responses of antigen presenting cells, including those of dendritic cells reducing the expression of IL-12 [22, 23]. Together these findings emphasize the role of this new super-family of autacoids in the maintenance of homeostasis and re-establishment of function following challenge, whether this is sterile or infective in nature.

**Table 1:** Lipid mediator metabolomes, abbreviations and complete stereochemistry.

Metabolome	Mediator	Abbreviation	Stereochemistry
DHA	Resolvin D1	RvD1	7*S*, 8*R*, 17*S*-trihydroxy-4*Z*, 9*E*, 11*E*, 13*Z*, 15*E*, 19*Z*-docosahexaenoic acid
	Resolvin D2	RvD2	7*S*, 16*R*, 17*S*-trihydroxy-4*Z*, 8*E*, 10*Z*, 12*E*, 14*E*, 19*Z*-docosahexaenoic acid
	Resolvin D3	RvD3	4*S*, 11*R*, 17*S*-trihydroxy-5*Z*, 7*E*, 9*E*, 13*Z*, 15*E*, 19Z-docosahexanoic acid
	Resolvin D4	RvD4	4*S*, 5*R*, 17*S*-trihydroxy-6*E*, 8*E*, 10*Z*, 13*Z*, 15*E*, 19*Z*-docosahexaenoic acid
	Resolvin D5	RvD5	7*S*, 17*S*-dihydroxy-4*Z*, 8*E*, 10*Z*, 13*Z*, 15*E*, 19*Z*-docosahexaenoic acid
	Maresin 1	MaR1	7*R*, 14*S*-dihydroxy-4*Z*, 8*E*, 10*E*, 12*Z*, 16*Z*, 19*Z*-docosahexaenoic acid
	Protectin conjugate in tissue regeneration 1	PCTR1	16*R*-glutathionyl, 17*S*-hydroxy-4*Z*, 7*Z*, 10*Z*, 12*E*, 14*E*, 19*Z*-docosahexaenoic acid
	Protectin conjugate in tissue regeneration 2	PCTR2	16-cysteinylglycinyl, 17*S*-hydroxy-4*Z*, 7*Z*, 10*Z*, 12, 14, 19*Z*-docosahexaenoic acid
	Protectin conjugate in tissue regeneration 3	PCTR3	16-cysteinyl, 17*S*-hydroxy-4*Z*, 7*Z*, 10*Z*, 12, 14, 19*Z*-docosahexaenoic acid

EPA	Resolvin E1	RvE1	5*S*, 12*R*, 18*R*-trihydroxy-6*Z*, 8*E*, 10*E*, 14*Z*, 16*E*-eicosapentaenoic acid

AA	Lipoxin A_4_	LXA_4_	5*S*, 6*R*, 15*S*-trihydroxy-7*E*, 9*E*, 11*Z*, 13*E*-eicosatetraenoic acid
	Leukotriene B_4_	LTB_4_	5*S*, 12*R*-dihydroxy-6*Z*, 8*E*, 10*E*, 14*Z*-eicosatetraenoic acid
	Prostaglandin E_2_	PGE_2_	9-oxo-11*R*, 15*S*-dihydroxy-5*Z*, 13*E*-prostadienoic acid

Several mechanisms are now implicated in the regulation of SPM biosynthesis; regulation of micro RNA 219 controls 5-LOX expression [24]. Post-translation modification of 5-LOX leads to a switch in the product profile of the enzyme from the pro-inflammatory leukotriene (LT) B_4_ to the pro-resolving mediator LXA_4_ [25]. Sex hormones also play an important role in regulating SPM production, an action that is also tissue dependent, where for example in murine systems estrogen down-regulates LXA_4_ production in the eye [17]. In humans this hormone is associated with an increased derman and systemic SPM production including RvD, LX and RvE [26]. Recent results also demonstrate that the nervous system is essential in controlling local SPM formation, during both infectious and sterile inflammation [3, 5]. The present review highlights the recent evidence underpinning the role of the nervous system in regulating tissue SPM production and the role of this response in controlling innate immune responses to both sterile and infectious insults.

## Neuronal regulation of tissue lipid mediator profiles

Increasing evidence indicates that the initial response by resident leukocytes to both injury and infections has a significant bearing on outcome of the ensuing inflammation and whether this resolves or becomes chronic [27]. Given the roles that the vagus nerve plays in regulating host immune responses [1, 8, 9], we recently queried whether this nerve was also involved in the tempering of tissue leukocyte phenotype and responses. Using a systematic approach we found that cervical disruption of the vagus nerve lead to a shift in peritoneal concentrations of both pro-resolving as well as inflammation initiating eicosanoids [3]. Peritoneal prostaglandin concentrations were increased in vagotomised mice whereas concentrations of several pro-resolving mediators including the recently uncovered immunoresolvents Protectin Conjugates in Tissue Regeneration (PCTR) were decreased. Of note, cervical disruption of the left vagus did not significantly perturb peritoneal PCTR levels suggesting that the neuro-immune plexus was in this organ was associated with the right vagus. These changes in peritoneal lipid mediator concentrations point to a significant alteration in the ability of the host to mount a protective immune response given the increase in inflammation-initiating eicosanoids and a decrease in the tissue protective mediators; a lipid mediator profile associated with delayed/non-resolving inflammation [10, 28, 29].

## Vagus regulates PCTR biosynthesis in peritoneal ILC-3 and macrophages

Changes in peritoneal levels of these mediators were also associated with an alteration peritoneal leukocyte composition, phenotype and functions. Loss of vagal signaling impacted group 3 innate lymphoid cells (ILC-3). The observation that ILC-3 numbers were significantly decreased in the peritoneum of vagotomised mice demonstrates that the biological actions of these cells are not solely restricted to the gastro-intestinal epithelium. Our studies demonstrated that these cells are also found in milky spots within the greater omenutm in proximity to macrophages as well as neuronal bodies arising from the gastroepiploic nerve; a nerve that is linked to the right vagus nerve via the gastroepiploic plexus [30]. This highlights a role for neuronal signaling in controlling ILC-3 trafficking to organs, given the decreased peritoneal cell counts observed following resection of the vagal trunk [3]. These findings are in accord with recent studies demonstrating a role of the neuronal system in regulating ILC-3 responses. Disruption of vagal signaling also impacted the peritoneal macrophage population. Peritoneal macrophage numbers were increased in vagotomise mice, with a shift in both the expression of lineage markers, including arginease-1 and major histocompatibility complex II, as well as lipid mediator profiles. Of note, the biosynthesis of PCTRs in these peritoneal macrophages was altered indicating that vagal signaling controls macrophage lipid mediator biosynthesis. The role of ILC-3 in regulating both peritoneal PCTR concentrations as well as macrophage phenotype was highlighted by the observation that both were altered in Rag1^−/−^ mice when ILC-3 were depleted using an anti-CD90 antibody. In both WT and Rag1^−/−^ mice loss of ILC-3 also lead to a delay in resolution responses to *Escherichia coli* infections and an impaired ability of peritoneal leukocytes to clear bacteria [3]. Recent studies have also demonstrated an interaction between components of the neuronal system and ILC-3 [31, 32] as well as other immune cells including macrophages [33, 34] in controlling host response to both sterile and infectious insults in the gut mucosa. Thus these findings underscore a central role for neuronal control of immune responses in regulating in the maintenance of tissue homeostasis and the re-establishment of function following an inflammatory episode.

**Figure 1:**
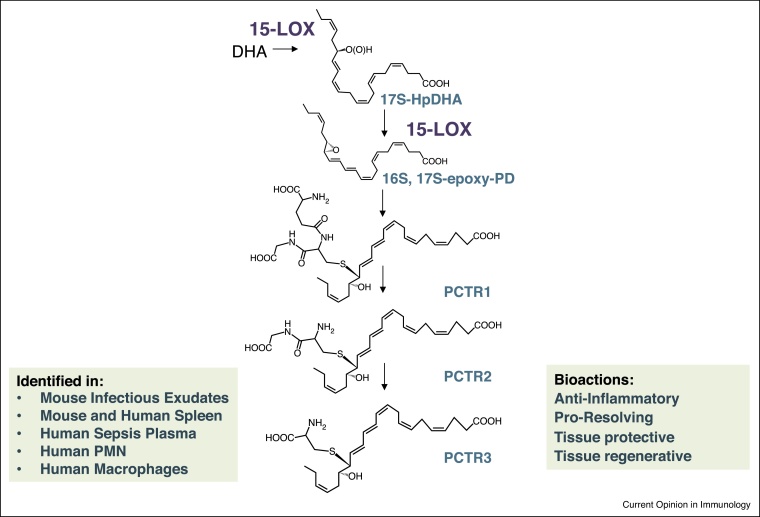
The PCTR biosynthetic pathway and biological actions. In the production of PCTRs DHA is oxygenated at carbon 17 to yield 17S-hydro(peroxy)-4Z,7Z,10Z,13Z,15E,19Z-docosahexaenoic acid. This is subsequently converted to an allylic epoxide intermediate via 15-LOX mediated abstraction of hydrogen from carbon 14 yielding 16S,17S-epoxy-4Z,7Z,10Z,12E,14E,19Z-docosahexaenoic acid (16S, 17S-epoxy-PD). In murine systems these reactions are catalyzed by the 12/15-LOX exnyme. The epoxide is substrate for conversion to the peptide–lipid conjugated mediator 16R-glutathionyl,17S-hydroxy-4Z,7Z,10Z,12E,14E,19Z-docosahexaenoic acid (PCTR1) that is precursor to 16-cysteinylglycinyl,17S-hydroxy-4Z,7Z,10Z,12,14,19Z-docosahexaenoic acid (PCTR2) which in turn is precursor to 16-cysteinyl,17S-hydroxy-4Z,7Z,10Z,12,14,19Z-docosahexaenoic acid (PCTR3). The PCTRs carry potent biological actions in promoting the resolution of infectious inflammation by regulating leukocyte trafficking and the uptake and killing of bacteria as well as tissue repair and regeneration.

In the lamina propria ILC-3 are central in regulating tissue macrophage responses by producing regulatory factors such as GM-CSF, a mechanism that can be either protective [35] or pathogenic [36]. These cells also express 15-LOX type 1 (mouse cells express the homologous enzyme: 12/15-LOX), the initiating enzyme in the PCTR biosynthetic pathway, and the activity of this enzyme is upregulated by the neurotransmitter acetylcholine [3]. PCTRs are a newly uncovered family of pro-resolving mediators that control neutrophil and macrophage responses to both sterile and infectious stimuli. This family of mediators is biosynthesized via the insertion of molecular oxygen by 15-liopxygenase at carbon 17 of DHA to yield 17S-hydro(peroxy)-4Z,7Z,10Z,13Z,15E,19Z-docosahexaenoic acid. This is subsequently converted to an allylic epoxide intermediate via 15-LOX mediated abstraction of hydrogen from carbon 14 yielding 16S, 17S-epoxy-4Z,7Z,10Z,12E,14E,19Z-docosahexaenoic acid (16S, 17S-epoxy-PD; Figure 1). The epoxide is substrate for conversion by glutathione-S-transferases to the peptide–lipid conjugated mediator 16R-glutathionyl, 17S-hydroxy-4Z,7Z,10Z,12E,14E,19Z-docosahexaenoic acid (PCTR1) that is precursor to 16-cysteinylglycinyl, 17S-hydroxy-4Z,7Z,10Z,12,14,19Z-docosahexaenoic acid (PCTR2) and 16-cysteinyl, 17S-hydroxy-4Z,7Z,10Z,12,14,19Z-docosahexaenoic acid (PCTR3). Each of these mediators promotes the phagocytosis of bacteria by neutrophils and macrophages as well as macrophage efferocytosis [37, 38, 39]. PCTR1 downregulates the production of inflammatory cytokines by macrophages including tumor necrosis factor-α, Interleukin (IL)-3, IL-8, and IL-12(p40) and regulate leukocyte trafficking increasing monocyte/macrophage trafficking and limiting neutrophil recruitment to the site of inflammation [39]. In addition, each of the PCTRs accelerates tissue regeneration in planaria [37, 38].

**Figure 2:**
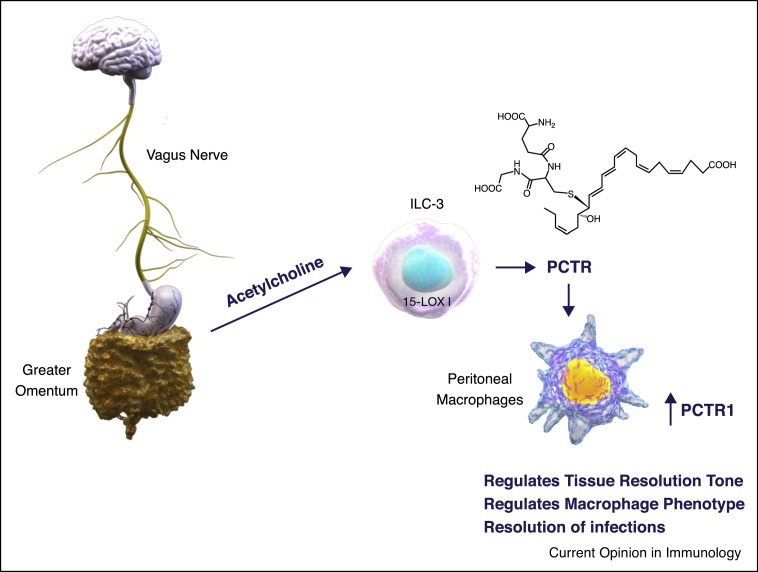
PCTR mediate neuronal control of tissue resolution tone. Neuronal singling in the peritoneum regulates ILC-3 PCTR production that controls macrophage phenotype, upregulating macrophage SPM biosynthesis and regulating host responses to bacterial infection.

**Figure 3:**
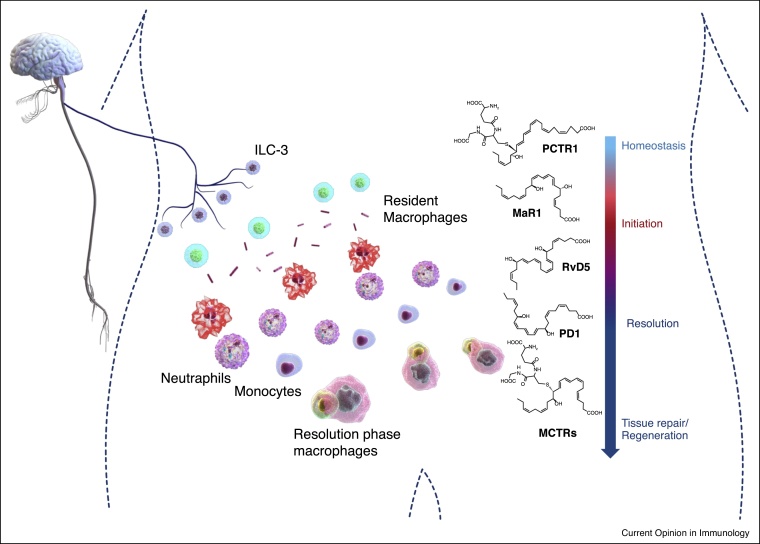
Temporal regulation of exudate SPM biosynthesis during self-limited infections. Under ideal conditions neuronal signal regulates tissue PCTR biosynthesis. This controls tissue resident macrophage phenotype and function. Infectious challenge activates resident leukocytes, primarily macrophages, and upregulates maresin (MaR)1 biosynthesis, this mediator promotes the uptake and clearance of apoptotic cells and bacteria and antagonizes the signaling of the leukocyte chemoattractant LTB_4_ by acting as a partial agonist/antagonist to the LTB_4_ receptor (BLT1). This regulatory action is also shared with the MaR1 further metabolite 22-OH-MaR1 [43]. As inflammatory exudate matures neutrophil numbers reach a maximum and a shift in the lipid mediator profile occurs with an upregulation in RvD5 and PD1 concentrations that denotes the onset of resolution [13]. This shift in lipid mediator profile is also associated with an increased recruitment of monocytes to the peritoneum and an increase in the number of resolution phase macrophages. During these late stages of inflammation-resolution there is also the formation of tissue-reparative and regenerative mediators termed as maresin conjugates in tissue regeneration (MCTRs) [15]. The biosynthesis of these mediators in human macrophages is initiated via the 14-lipoxygenation of DHA, to yield three novel peptide–lipid conjugated mediators namely MCTR1, MCTR2 and MCTR3 [15].

Given that leukocytes are central in SPM biosynthesis, loss of PCTRs in mice following the disruption of peritoneal neuronal signaling indicates that the neuro-immune plexus is essential for controlling PCTR biosynthesis in peritoneal leukocytes. In this context production of SPM, in particular PCTR1, by ILC-3 is central in regulating peritoneal macrophage phenotype and responses whereby co-incubations of peritoneal macrophages with either ILC-3 or PCTR1 restores their lipid mediator profiles and their ability to uptake bacteria (Figure 2). This was of biological relevance in infections since reconstitution of vagotomised mice with either ILC-3 or PCTR1 also lead to a restoration of host responses following *E. coli* inoculation, significantly reducing the number of leukocytes recruited to the peritoneal cavity, shortening the resolution interval (i.e. the time it takes for recruited neutrophils to traffic out of the peritoneum) and upregulated the ability of peritoneal leukocytes to phagocytose and kill bacteria [3]. Furthermore, it rectified LM-SPM biosynthesis during the course of the inflammatory response. Given the role that different SPM play in regulating distinct aspects of the host immune response during select stages of infectious-inflammation this is critical for the re-establishment of function (see [28] for a detailed review and Figure 3). Therefore these findings underscore the role of ILC-3 in orchestrating peritoneal macrophage responses and tissue resolution tone. They also highlight that dysregulation in SPM production alters leukocyte phenotype and consequently function.

## SPM regulation by the vagus in sterile inflammation

The regulation of lipid mediators by neuronal circuits is also central in the control of inflammation to sterile injury. The vagus nerve regulates the expression of the axonal guidance molecule netrin 1, a protein that in addition to regulating neuronal development also controls immune responses [5, 40, 41]. Incubation of netrin 1 with primary human monocytes upregulates the biosynthesis of several pro-resolving mediators including RvD1 and LXA_4_. Netrin 1 also increases SPM production *in vivo* during acute-self limited inflammation, were this protein upregulates exudate RvD5 and PD1 concentrations during zymosan initiated peritonitis. Loss of netrin 1 expression in both vagotomized mice and netrin 1 heterozygote mice lead to dysregulated LM production following sterile challenged, including a reduction in exudate RvD1 and an increase in the inflammation initiating eicosanoids LTB_4_ and PGE_2_ [5]. This was also associated with a disruption of resolution processes, increased neutrophil recruitment to the peritoneum, delayed resolution of inflammation and an increase in the exudate levels of several pro-inflammatory cytokines including TNF-α, IL-6 and MIP-1α [5]. Of note, netrin 1 also regulates macrophage efferocytosis, an action that was dependent on the ability of these cells to produce SPM, given that this action was lost in macrophages from 12/15-LOX deficient mice (see Figure 2). In addition, administration of RvD1 to netrin deficient mice restored resolution responses, reducing the amplitude as well as the duration of the inflammatory response [5].

## Concluding remarks

The biological actions of SPM are now appreciated to be relevant to many experimental as well as human settings where they regulate the cardinal signs of resolution that include *remotio infectiones agens* (clearance of infective agents), *remotio obruta* (clearance of debris), *avaritiam ex munere* (gain of function) and *indolentia* (analgesia) [37, 38]. Dysregulation in the production of these mediators is also associated with the development of disease [11, 12, 14, 29, 37, 38, 42] with the mechanisms leading to this impaired production remaining of interest. Thus, the appreciation that the neuro-immune synapse is important in regulating tissue levels of these mediators, with neurotransmitters such acetylcholine being important in controlling local SPM biosynthesis, may provide new leads in the understanding of disease mechanisms. Moreover, these new links to neural immune networks represent new leads in the development of approaches that harness the potent biological actions of SPM and do not carry the many side effects associated with current therapeutics.

## Conflict of interest

The authors declare that they have no competing interests.

## References

[1] Chavan S.S., Pavlov V.A., Tracey K.J. (2017). Mechanisms and therapeutic relevance of neuro-immune communication. Immunity.

[2] Sun J., Singh V., Kajino-Sakamoto R., Aballay A. (2011). Neuronal GPCR controls innate immunity by regulating noncanonical unfolded protein response genes. Science.

[3] Dalli J., Colas R.A., Arnardottir H., Serhan C.N. (2017). Vagal regulation of group 3 innate lymphoid cells and the immunoresolvent PCTR1 controls infection resolution. Immunity.

[4] Dubeykovskaya Z., Si Y., Chen X., Worthley D.L., Renz B.W., Urbanska A.M., Hayakawa Y., Xu T., Westphalen C.B., Dubeykovskiy A. (2016). Neural innervation stimulates splenic TFF2 to arrest myeloid cell expansion and cancer. Nat Commun.

[5] Mirakaj V., Dalli J., Granja T., Rosenberger P., Serhan C.N. (2014). Vagus nerve controls resolution and pro-resolving mediators of inflammation. J Exp Med.

[6] Li T., Zuo X., Zhou Y., Wang Y., Zhuang H., Zhang L., Zhang H., Xiao X. (2010). The vagus nerve and nicotinic receptors involve inhibition of HMGB1 release and early pro-inflammatory cytokines function in collagen-induced arthritis. J Clin Immunol.

[7] Willemze R.A., Welting O., van Hamersveld H.P., Meijer S.L., Folgering J.H.A., Darwinkel H., Witherington J., Sridhar A., Vervoordeldonk M.J., Seppen J. (2017). Neuronal control of experimental colitis occurs via sympathetic intestinal innervation. Neurogastroenterol Motil.

[8] Huston J.M., Ochani M., Rosas-Ballina M., Liao H., Ochani K., Pavlov V.A., Gallowitsch-Puerta M., Ashok M., Czura C.J., Foxwell B. (2006). Splenectomy inactivates the cholinergic antiinflammatory pathway during lethal endotoxemia and polymicrobial sepsis. J Exp Med.

[9] Koopman F.A., Chavan S.S., Miljko S., Grazio S., Sokolovic S., Schuurman P.R., Mehta A.D., Levine Y.A., Faltys M., Zitnik R. (2016). Vagus nerve stimulation inhibits cytokine production and attenuates disease severity in rheumatoid arthritis. Proc Natl Acad Sci U S A.

[10] Serhan C.N. (2017). Discovery of specialized pro-resolving mediators marks the dawn of resolution physiology and pharmacology. Mol Aspects Med.

[11] Serhan C.N., Chiang N., Dalli J. (2015). The resolution code of acute inflammation: novel pro-resolving lipid mediators in resolution. Semin Immunol.

[12] Serhan C.N., Dalli J., Colas R.A., Winkler J.W., Chiang N. (2015). Protectins and maresins: new pro-resolving families of mediators in acute inflammation and resolution bioactive metabolome. Biochim Biophys Acta.

[13] Chiang N., Fredman G., Backhed F., Oh S.F., Vickery T., Schmidt B.A., Serhan C.N. (2012). Infection regulates pro-resolving mediators that lower antibiotic requirements. Nature.

[14] Bohr S., Patel S.J., Sarin D., Irimia D., Yarmush M.L., Berthiaume F. (2013). Resolvin D2 prevents secondary thrombosis and necrosis in a mouse burn wound model. Wound Repair Regen.

[15] Dalli J., Chiang N., Serhan C.N. (2014). Identification of 14-series sulfido-conjugated mediators that promote resolution of infection and organ protection. Proc Natl Acad Sci U S A.

[16] Hasturk H., Kantarci A., Goguet-Surmenian E., Blackwood A., Andry C., Serhan C.N., Van Dyke T.E. (2007). Resolvin E1 regulates inflammation at the cellular and tissue level and restores tissue homeostasis in vivo. J Immunol.

[17] Gao Y., Min K., Zhang Y., Su J., Greenwood M., Gronert K. (2015). Female-specific downregulation of tissue polymorphonuclear neutrophils drives impaired regulatory T cell and amplified effector T cell responses in autoimmune dry eye disease. J Immunol.

[18] Chiurchiu V., Leuti A., Dalli J., Jacobsson A., Battistini L., Maccarrone M., Serhan C.N. (2016). Proresolving lipid mediators resolvin D1, resolvin D2, and maresin 1 are critical in modulating T cell responses. Sci Transl Med.

[19] Ramon S., Baker S.F., Sahler J.M., Kim N., Feldsott E.A., Serhan C.N., Martinez-Sobrido L., Topham D.J., Phipps R.P. (2014). The specialized proresolving mediator 17-HDHA enhances the antibody-mediated immune response against influenza virus: a new class of adjuvant?. J Immunol.

[20] Lima-Garcia J.F., Dutra R.C., da Silva K., Motta E.M., Campos M.M., Calixto J.B. (2011). The precursor of resolvin D series and aspirin-triggered resolvin D1 display anti-hyperalgesic properties in adjuvant-induced arthritis in rats. Br J Pharmacol.

[21] Xu Z.Z., Zhang L., Liu T., Park J.Y., Berta T., Yang R., Serhan C.N., Ji R.R. (2010). Resolvins RvE1 and RvD1 attenuate inflammatory pain via central and peripheral actions. Nat Med.

[22] Arita M., Bianchini F., Aliberti J., Sher A., Chiang N., Hong S., Yang R., Petasis N.A., Serhan C.N. (2005). Stereochemical assignment, antiinflammatory properties, and receptor for the omega-3 lipid mediator resolvin E1. J Exp Med.

[23] Sawada Y., Honda T., Hanakawa S., Nakamizo S., Murata T., Ueharaguchi-Tanada Y., Ono S., Amano W., Nakajima S., Egawa G. (2015). Resolvin E1 inhibits dendritic cell migration in the skin and attenuates contact hypersensitivity responses. J Exp Med.

[24] Recchiuti A., Krishnamoorthy S., Fredman G., Chiang N., Serhan C.N. (2011). MicroRNAs in resolution of acute inflammation: identification of novel resolvin D1-miRNA circuits. FASEB J.

[25] Fredman G., Ozcan L., Spolitu S., Hellmann J., Spite M., Backs J., Tabas I. (2014). Resolvin D1 limits 5-lipoxygenase nuclear localization and leukotriene B4 synthesis by inhibiting a calcium-activated kinase pathway. Proc Natl Acad Sci U S A.

[26] Rathod K.S., Kapil V., Velmurugan S., Khambata R.S., Siddique U., Khan S., Van Eijl S., Gee L.C., Bansal J., Pitrola K. (2017). Accelerated resolution of inflammation underlies sex differences in inflammatory responses in humans. J Clin Invest.

[27] Serhan C.N., Savill J. (2005). Resolution of inflammation: the beginning programs the end. Nat Immunol.

[28] Dalli J. (2017). Does promoting resolution instead of inhibiting inflammation represent the new paradigm in treating infections?. Mol Aspects Med.

[29] Fredman G., Spite M. (2017). Specialized pro-resolving mediators in cardiovascular diseases. Mol Aspects Med.

[30] Swanson L.W. (2015). Neuroanatomical Terminology: A Lexicon of Classical Origins and Historical Foundations.

[31] Ibiza S., Garcia-Cassani B., Ribeiro H., Carvalho T., Almeida L., Marques R., Misic A.M., Bartow-McKenney C., Larson D.M., Pavan W.J. (2016). Glial-cell-derived neuroregulators control type 3 innate lymphoid cells and gut defence. Nature.

[32] Klose C.S., Artis D. (2016). Innate lymphoid cells as regulators of immunity, inflammation and tissue homeostasis. Nat Immunol.

[33] Gabanyi I., Muller P.A., Feighery L., Oliveira T.Y., Costa-Pinto F.A., Mucida D. (2016). Neuro-immune interactions drive tissue programming in intestinal macrophages. Cell.

[34] Muller P.A., Koscso B., Rajani G.M., Stevanovic K., Berres M.L., Hashimoto D., Mortha A., Leboeuf M., Li X.M., Mucida D. (2014). Crosstalk between muscularis macrophages and enteric neurons regulates gastrointestinal motility. Cell.

[35] Mortha A., Chudnovskiy A., Hashimoto D., Bogunovic M., Spencer S.P., Belkaid Y., Merad M. (2014). Microbiota-dependent crosstalk between macrophages and ILC3 promotes intestinal homeostasis. Science.

[36] Pearson C., Thornton E.E., McKenzie B., Schaupp A.L., Huskens N., Griseri T., West N., Tung S., Seddon B.P., Uhlig H.H. (2016). ILC3 GM-CSF production and mobilisation orchestrate acute intestinal inflammation. Elife.

[37] Dalli J., Chiang N., Serhan C.N. (2015). Elucidation of novel 13-series resolvins that increase with atorvastatin and clear infections. Nat Med.

[38] Dalli J., Ramon S., Norris P.C., Colas R.A., Serhan C.N. (2015). Novel proresolving and tissue-regenerative resolvin and protectin sulfido-conjugated pathways. FASEB J.

[39] Ramon S., Dalli J., Sanger J.M., Winkler J.W., Aursnes M., Tungen J.E., Hansen T.V., Serhan C.N. (2016). The protectin PCTR1 is produced by human M2 macrophages and enhances resolution of infectious inflammation. Am J Pathol.

[40] Ramkhelawon B., Hennessy E.J., Menager M., Ray T.D., Sheedy F.J., Hutchison S., Wanschel A., Oldebeken S., Geoffrion M., Spiro W. (2014). Netrin-1 promotes adipose tissue macrophage retention and insulin resistance in obesity. Nat Med.

[41] Rosenberger P., Schwab J.M., Mirakaj V., Masekowsky E., Mager A., Morote-Garcia J.C., Unertl K., Eltzschig H.K. (2009). Hypoxia-inducible factor-dependent induction of netrin-1 dampens inflammation caused by hypoxia. Nat Immunol.

[42] Barden A.E., Moghaddami M., Mas E., Phillips M., Cleland L.G., Mori T.A. (2016). Specialised pro-resolving mediators of inflammation in inflammatory arthritis. Prostaglandins Leukot Essent Fatty Acids.

[43] Colas R.A., Dalli J., Chiang N., Vlasakov I., Sanger J.M., Riley I.R., Serhan C.N. (2016). Identification and actions of the Maresin 1 metabolome in infectious inflammation. J Immunol.

